# Involvement of MAPK/NF-κB Signaling in the Activation of the Cholinergic Anti-Inflammatory Pathway in Experimental Colitis by Chronic Vagus Nerve Stimulation

**DOI:** 10.1371/journal.pone.0069424

**Published:** 2013-08-02

**Authors:** Peng Sun, Kewen Zhou, Sheng Wang, Ping Li, Sijuan Chen, Guiping Lin, Yan Zhao, Tinghuai Wang

**Affiliations:** Department of Physiology, Zhongshan School of Medicine, Sun Yat-sen University, Guangzhou, People’s Republic of China; University of California, Los Angeles, United States of America

## Abstract

**Background:**

Autonomic nervous system dysfunction is implicated in the etiopathogenesis of inflammatory bowel diseases (IBD). Therapies that increase cardiovagal activity, such as Mind-Body interventions, are currently confirmed to be effective in clinical trials in IBD. However, a poor understanding of pathophysiological mechanisms limits the popularization of therapies in clinical practice. The aim of the present study was to explore the mechanisms of these therapies against 2,4,6-trinitrobenzenesulfonic acid (TNBS)-induced colitis in rats using a chronic vagus nerve stimulation model *in vivo*, as well as the lipopolysaccharide (LPS)-induced inflammatory response in human epithelial colorectal adenocarcinoma cells (Caco-2) by acetylcholine *in vitro*.

**Methods and Results:**

Colitis was induced in rats with rectal instillation of TNBS, and the effect of chronic VNS (0.25 mA, 20 Hz, 500 ms) on colonic inflammation was evaluated. Inflammatory responses were assessed by disease activity index (DAI), histological scores, myeloperoxidase (MPO) activity, inducible nitric oxide synthase (iNOS), TNF-α and IL-6 production. The expression of Mitogen-activated protein kinases (MAPK) family members, IκB-α, and nuclear NF-κB p65 were studied by immunoblotting. Heart rate variability (HRV) analysis was also applied to assess the sympathetic-vagal balance. DAI, histological scores, MPO activity, iNOS, TNF-α and IL-6 levels were significantly decreased by chronic VNS. Moreover, both VNS and acetylcholine reduced the phosphorylation of MAPKs and prevented the nuclear translocation of NF-κB p65. Methyllycaconitine (MLA) only reversed the inhibitory effect on p-ERK and intranuclear NF-κB p65 expression by ACh *in vitro*, no significant change was observed in the expression of p-p38 MAPK or p-JNK by MLA.

**Conclusion:**

Vagal activity modification contributes to the beneficial effects of the cholinergic anti-inflammatory pathway in IBD-related inflamed colonic mucosa based on the activation of MAPKs and nuclear translocation of NF-κB. Our work may provide key pathophysiological mechanistic evidence for novel therapeutic strategies that increase the cardiovagal activity in IBD patients.

## Introduction

Inflammatory Bowel Diseases (IBD) are a group of chronic inflammatory disorders of the gastrointestinal (GI) tract that are subdivided into ulcerative colitis (UC) and Crohn’s disease (CD). The typical clinical manifestation of IBD consists of ulcerations in the intestinal mucosa and in CD, inflammation may span as the transmural pattern. Although the etiopathogenesis of IBD remains unknown, it is currently thought that many factors participate in the pathology of IBD, such as the overproduction of pro-inflammatory mediators including cytokines, dysfunction of the immune system, and an imbalance of microflora [1]. Among these factors, the overproduction of pro-inflammatory mediators, such as TNF-a, IL-1, IL-6, and IL-8, the presence of highly activated inflammatory cells such as macrophages neutrophils, and monocytes, as well as an excessive production of reactive oxygen species (ROS) appear to play major roles in the pathogenesis of IBD [2-7]. Salicylates, glucocorticoids and immunosuppressive agents, which are the dominant current therapies for IBD over the last few decades, are generally effective in either ulcerative colitis or Crohn’s disease. However, the marked deleterious effects still cannot be ignored. Nowadays, biological therapies such as Anti-TNF-alpha biologic compounds (e.g. Infliximab) alone or in combination with immunosuppressives have shown great efficacy in IBD and have been considered as the gold standard in IBD therapeutic strategy [8]. However, despite the striking effect of biological therapies, the increase of economic cost and the dependent on the medication indefinitely still bring great concern on the arrival of novel therapeutic strategies.

Complementary and alternative medicine (CAM), such as Mind-Body interventions including biofeedback, yoga, meditation, hypnotherapy and relaxation training, are widely used by patients with IBD and other gastrointestinal complaints. Despite the significant effects in both animal models and clinical trials as well as the benefit that CAM treatments are cost-effective for the patients, the lack of modern pathophysiological and pharmacological mechanisms and the dependence on practices make these interventions difficult to popularize [9]. However, recent studies have shown that central nervous system (CNS)-based behavioral therapy might inhibit the release of pro-inflammatory cytokines by increasing parasympathetic activity [10]. Our previous work also demonstrated that biofeedback therapy can significantly improve vagal tone and can inhibit sympathetic activity [11,12]. The results of heart rate variability (HRV) analysis have shown that patients with IBD exhibited a higher level of sympathetic activity and a lower level of parasympathetic activity than healthy subjects, and a growing body of evidence suggests the existence of autonomic nervous system (ANS) dysfunctions in patients with IBD [13-15], which may also depend on the psychological adjustment of the patients [16]. Therefore, we speculate that Mind-Body interventions such as biofeedback might protect against the development of IBD by modulating sympathovagal balance and improving the psychological adjustment and adaptability.

Recently, Tracey and colleagues reported the anti-inflammatory effect of vagus nerve stimulation on the systemic inflammatory response to endotoxin. They identiﬁed the neural mechanisms of the cholinergic anti-inflammatory pathway (CAP): vagal efferents innervate many of the organs associated with the immune system, including the heart, liver, and gastrointestinal system. Acetylcholine (ACh) released from the vagal efferents modulates immune responses via alpha 7 nicotinic receptors (α7nAchRs) on human macrophages that inhibit NF-κB and, consequently, cytokine (TNF-α, IL-1, etc.) synthesis and release [17-21]. The anti-inflammatory role of the cholinergic pathway has been demonstrated in experimental colitis using selective α7nAChR agonists [22], vagotomy [23,24] and VNS [25], but the mechanisms involved are unknown.

Mitogen-activated protein kinases (MAPKs) are implicated in a wide range of signaling cascades wherein various extracellular stimuli induce inﬂammation, including the production of inﬂammatory mediators, and these targets have naturally become the focus of attention in IBD research. There are three main components of the MAPK family, including the extracellular signal-regulated kinases (ERKs) (ERK1/2 or p42/p44), the c-Jun N-terminal kinases (JNKs) (JNK/SAPK) and the p38 MAPK [26,27]. Activation of these components can be independent of each other or overlapping, and the phosphorylation of particular amino acid sequences of MAPKs is required for their full activation. Activated MAPKs can then bind to and stimulate other kinase targets, translocate to the nucleus and activate the transcription of pro-inflammatory genes One of the well-studied downstream components of the MAPK signaling pathway is the nuclear transcription factor kappa B (NF-κB). The nuclear translocation of NF-κB is strongly activated by experimental colitis models, as well as in patients with IBD [28,29]. Similarly, α7nAchRs are thought to play an important role in gastrointestinal inflammation through an IκBα-dependent inhibition of NF-κB. Given this evidence, we suspected that MAPK/NF-κB signaling was involved in activation of the cholinergic anti-inflammatory pathway in IBD.

Based upon the anti-inflammatory effects of the cholinergic anti-inflammatory pathway, we assume that interventions enhancing cardiovagal modulation may ameliorate inflammatory responses in IBD. Accordingly, we established a chronic VNS model that imitated Mind-Body interventions to obtain an improved understanding of the anti-inflammatory response against 2,4,6-trinitrobenzenesulfonic acid (TNBS)-induced colitis in rats. Furthermore, we also applied HRV analysis to evaluate the sympathetic-vagal balance during experimental colitis. Finally, we studied the involvement of MAPKs and nuclear translocation of NF-κB signaling pathways in mounting the protective effect of chronic VNS or acetylcholine in vivo and in vitro, with the aim of providing pathophysiological mechanistic evidence for the therapeutic effectiveness of Mind-Body interventions.

## Results

### 1: Chronic VNS attenuates the severity of TNBS-induced colitis in rats

#### 1.1: Disease activity index (DAI)

**Figure 1:**
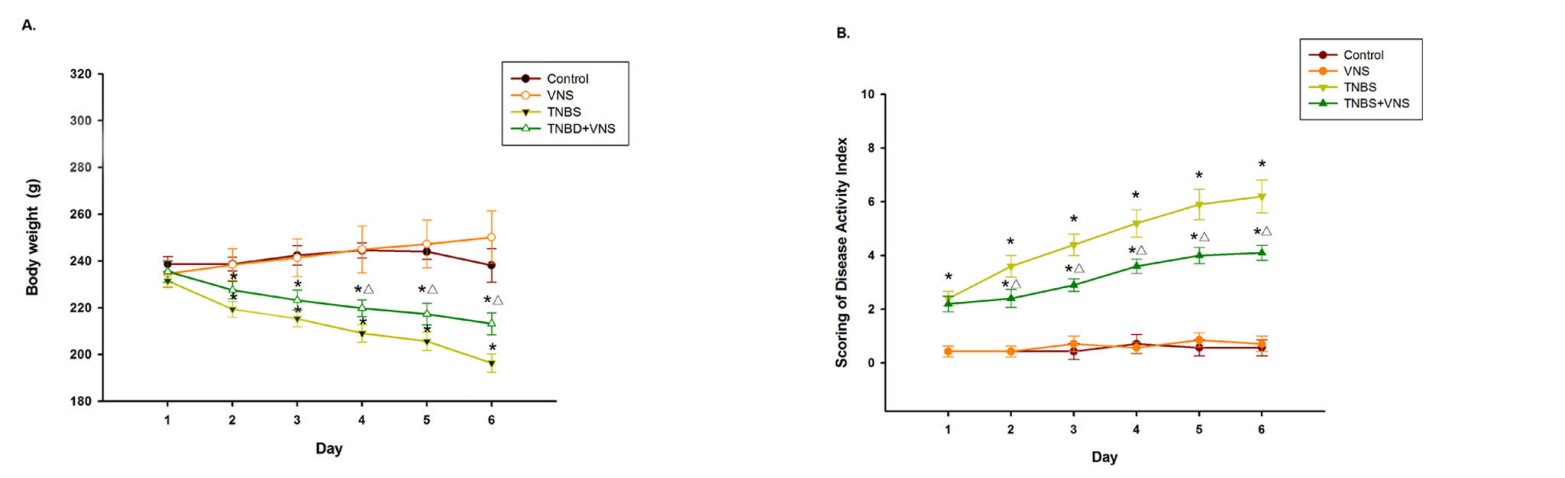
A-B. Time course of the disease activity index (DAI) (mean± SEM) scores and body weight (mean±SEM) in the control group (n=8), VNS group (n=8), TNBS group (n=10) or TNBS+VNS group (n=10). ∗ P<0.05 versus the control group and VNS group; △P<0.05 versus the TNBS group.

In rats with TNBS-induced colitis, bloody diarrhea, body weight loss, and reduced mobility were rapidly observed on the first day after TNBS injection, which resulted in a marked increase in the disease activity index (DAI) from day 1 onwards, compared with the control group (p<0.001). Chronic VNS significantly attenuated TNBS-induced clinical parameters from day 2 onwards compared with the TNBS group. Moreover, chronic VNS dramatically diminished the composed of DAI scores after rectal administration of TNBS (p<0.001) (Figure **1A-B**).

#### 1.2: Macroscopic and microscopic evaluation

**Figure 2:**
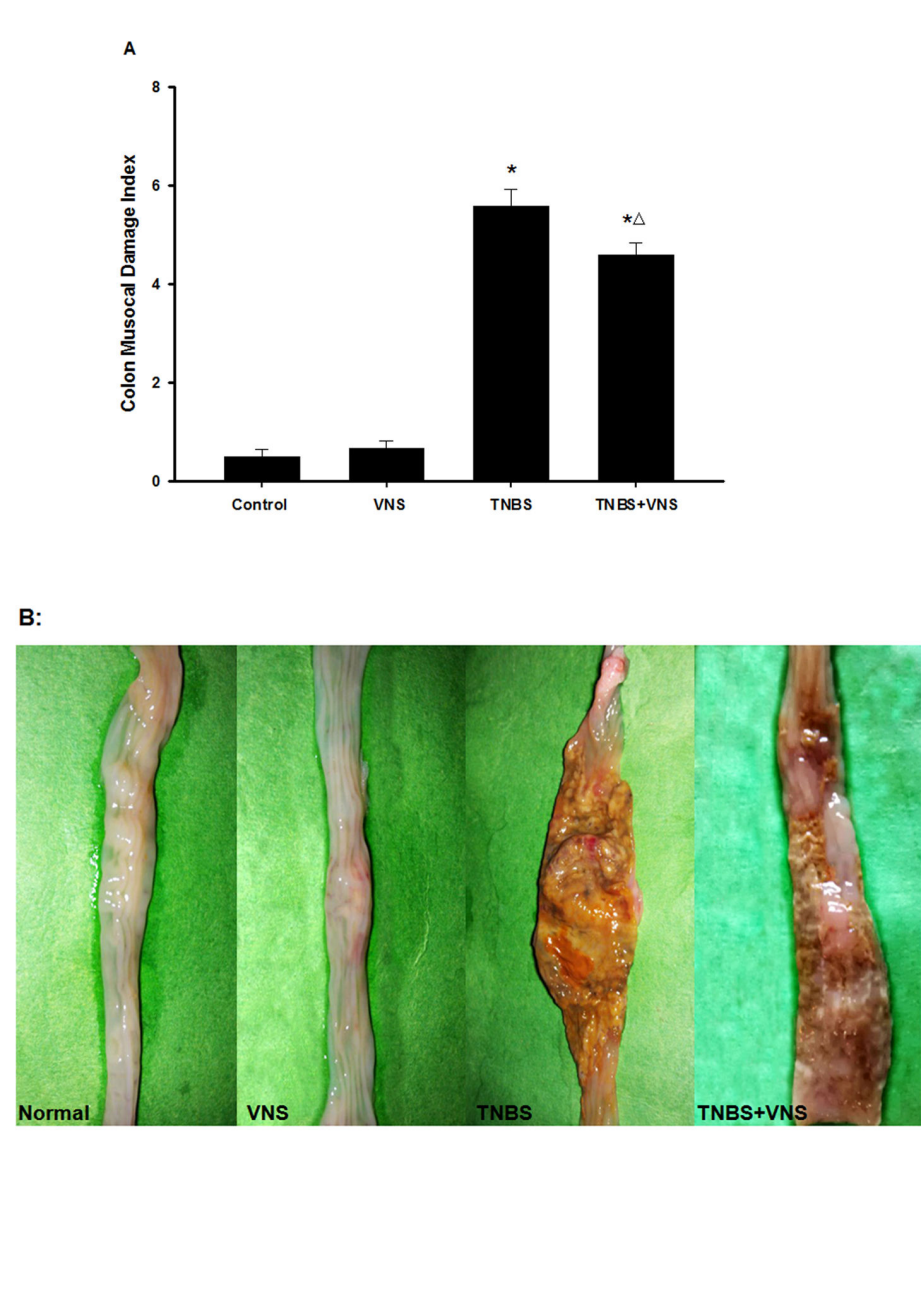
Chronic VNS reduces the severity of TNBS-induced colitis in rats. (A) Effects of administration of VNS after colonic instillation with TNBS; CMDI scores were quantiﬁed and expressed as the mean±SEM. (B) Representative macroscopic appearance of colonic mucosa across groups. * P<0.05 versus the control group and VNS group; △P<0.05 versus the TNBS group.

Notably, chronic VNS treatment in TNBS-rats ameliorated colon mucosa damage, including bowel wall thickening, dilation, edema, hyperemia, mucosal erosions, ulcers and adhesion to adjacent tissues (Figure **2A**). TNBS markedly increased the colon mucosal damage index (CMDI) scores compared with the control and VNS groups (p<0.01), whereas the TNBS+VNS group presented with a significant decrease in CMDI scores (p<0.01) (Figure **2B**).

**Figure 3:**
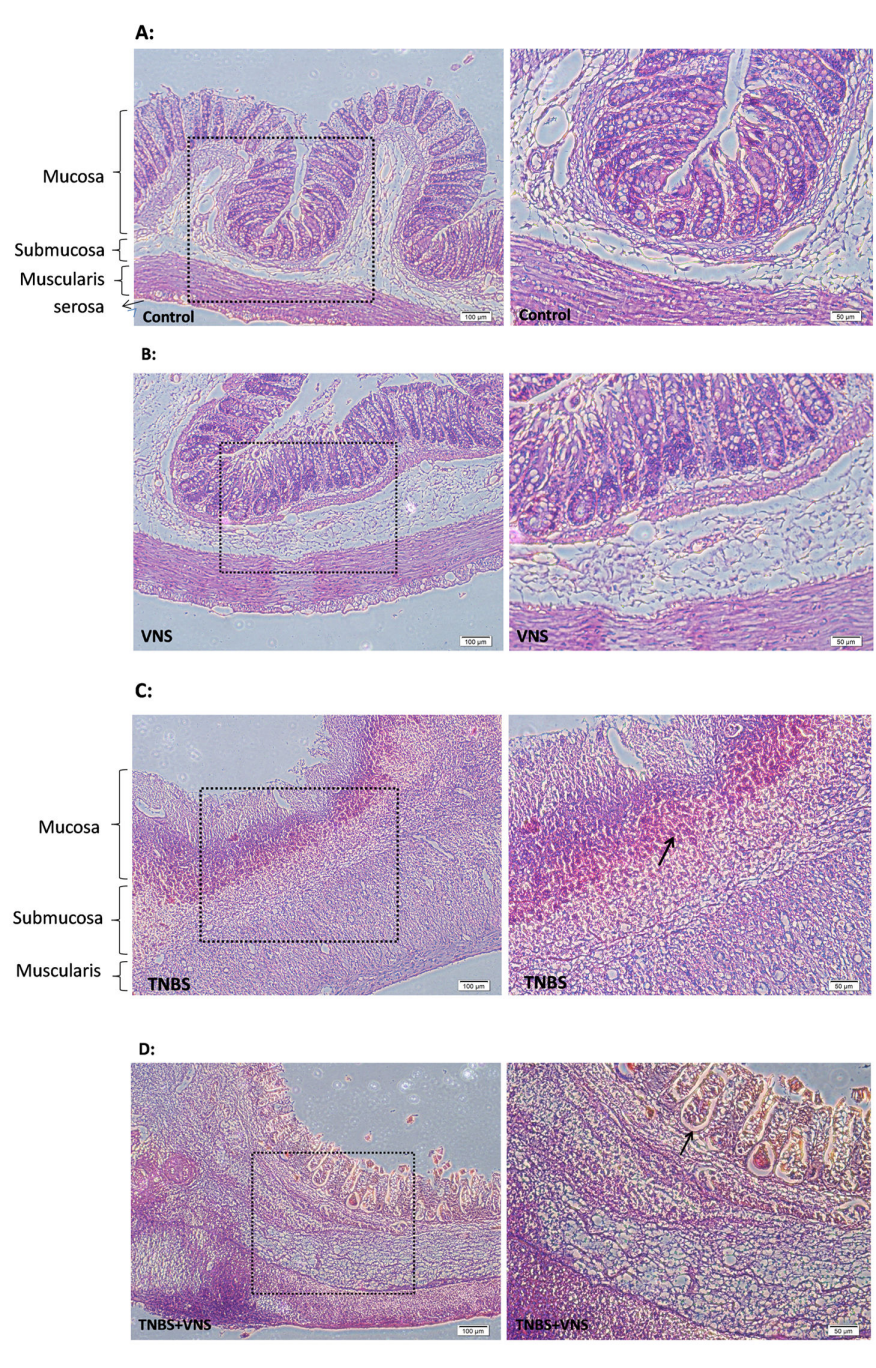
A–E. Chronic VNS ameliorates TNBS-induced colitis histologically. Photomicrographs (magnification ×100 & ×200) are representative of H&E stained slides of colonic tissues. A: Normal colonic mucosa of the SD rats. B: Mucosa of normal SD rats after VNS administration. C: Severe inflammation is present on the mucosa of TNBS-treated rats with inflammatory cell infiltration, ulcerations and goblet cell depletion (arrow). D: Treatment of TNBS-rats with chronic VNS for 6 days markedly decreased the inflammatory cell infiltration in the mucosa, and the arrow indicates the remaining goblet cells. E: The mean ± SEM of the histologic inflammatory scores are calculated for each group, as described in the methods section. * P<0.05 versus the control group; △P<0.05 versus the TNBS group.

As expected, the histological sections shown in Figure **3A–D** demonstrate that TNBS administration caused a severe inflammation that extended through the mucosa, muscularis mucosae and submucosa, including diffusion of granulocytes, leukomonocytes, inflammatory infiltrates, ulcerations, as well as goblet cell depletion. Therefore, the histological scores in the TNBS group were significantly increased in comparison with those of the control group (p<0.01), which exhibited normal mucosal structure. After 6 days of VNS administration, there was a pronounced reduction of pathomorphological signs of colonic damage, including the inhibition of inflammatory infiltration and ulcer healing, as well as the presence of goblet cells in the mucosal layer and a progressive restoration of the colonic architecture. The histological scores were significantly attenuated with chronic VNS compared with the TNBS group (p<0.05).

#### 1.3: Chronic VNS inhibits MPO and iNOS activity and decreases TNF-α and IL-6 levels in TNBS-induced colitis

**Figure 4:**
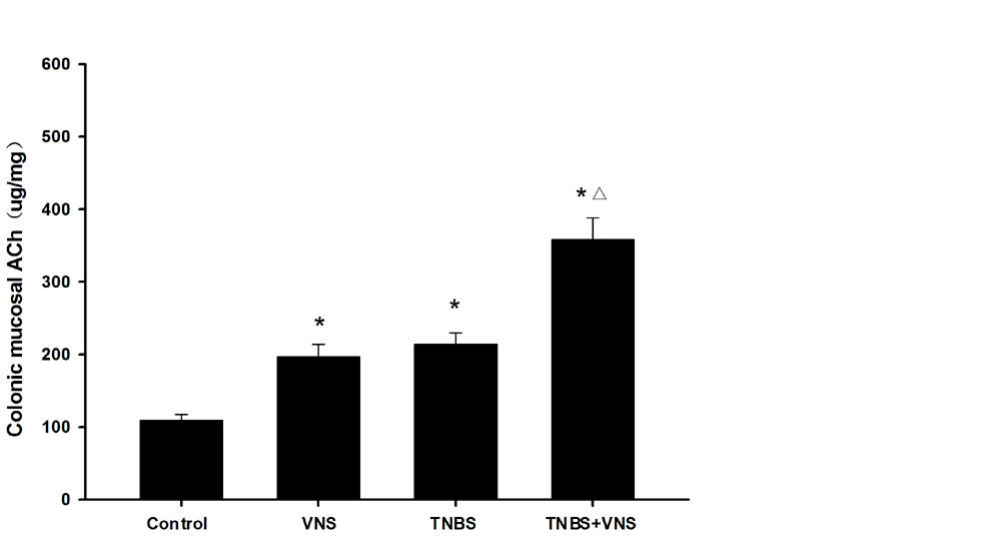
Colonic mucosal ACh level in the control group (n=8), VNS group (n=8), TNBS group (n=10) or TNBS+VNS group (n=10). * P<0.05 versus the control group; △P<0.05 versus the TNBS group.

Colonic damage by TNBS instillation was characterized by a significant increase of MPO and iNOS activity, which are indicators of neutrophil infiltration and oxidative stress injury. As shown in Table **1**, TNBS-treated rats with VNS administration demonstrated a remarkable decrease of MPO and iNOS activity (p<0.05). In addition, TNBS provoked a dramatic rise in the production of the pro-inflammatory cytokines TNF-α and IL-6, which were also inhibited in the TNBS+VNS group (p<0.05). Moreover, we also investigated mucosal ACh level during TNBS-induced colitis in this study. As shown in Figure **4**, colonic ACh level signiﬁcantly increased in TNBS group compared to the control group(p<0.05). VNS also markedly increased ACh level in colonic mucosa (p<0.05) and no significant difference was observed between VNS and TNBS group. However, ACh level in the TNBS+VNS group was higher than that in all other groups(p<0.05).

**Table 1:** Myeloperoxidase activity (MPO, U/ml), inducible nitric oxide synthase avtivity (iNOS, U/ml), tumour necrosis factor alpha (TNF-α, pg/mg tissue) and interleukin 6 (IL-6) levels after administration of chronic VNS in TNBS-rats.

Group	n	MPO (U/ml)	iNOS (U/ml)	TNF-a (pg/ml)	IL-6 (pg/ml)
Normal	8	1.76 ± 0.16	1.27 ± 0.75	98.31 ± 14.87	105.47 ± 13.56
VNS	8	1.89 ± 0.18	0.93 ± 0.23	101.53 ± 11.95	106.64 ± 11.47
TNBS	10	2.90 ± 0.36*	2.20 ± 1.05*	195.21 ± 10.76*	183.89 ± 9.77*
TNBS+VNS	10	2.09 ± 0.33*^#^	1.18 ± 0.58*^#^	151.21 ± 8.53*^#^	137.96 ± 8.04*^#^

Data are expressed as mean ± SEM. ∗P<0.05 versus control group; #P<0.05 versus TNBS group.

### 2: HRV analysis

**Figure 5:**
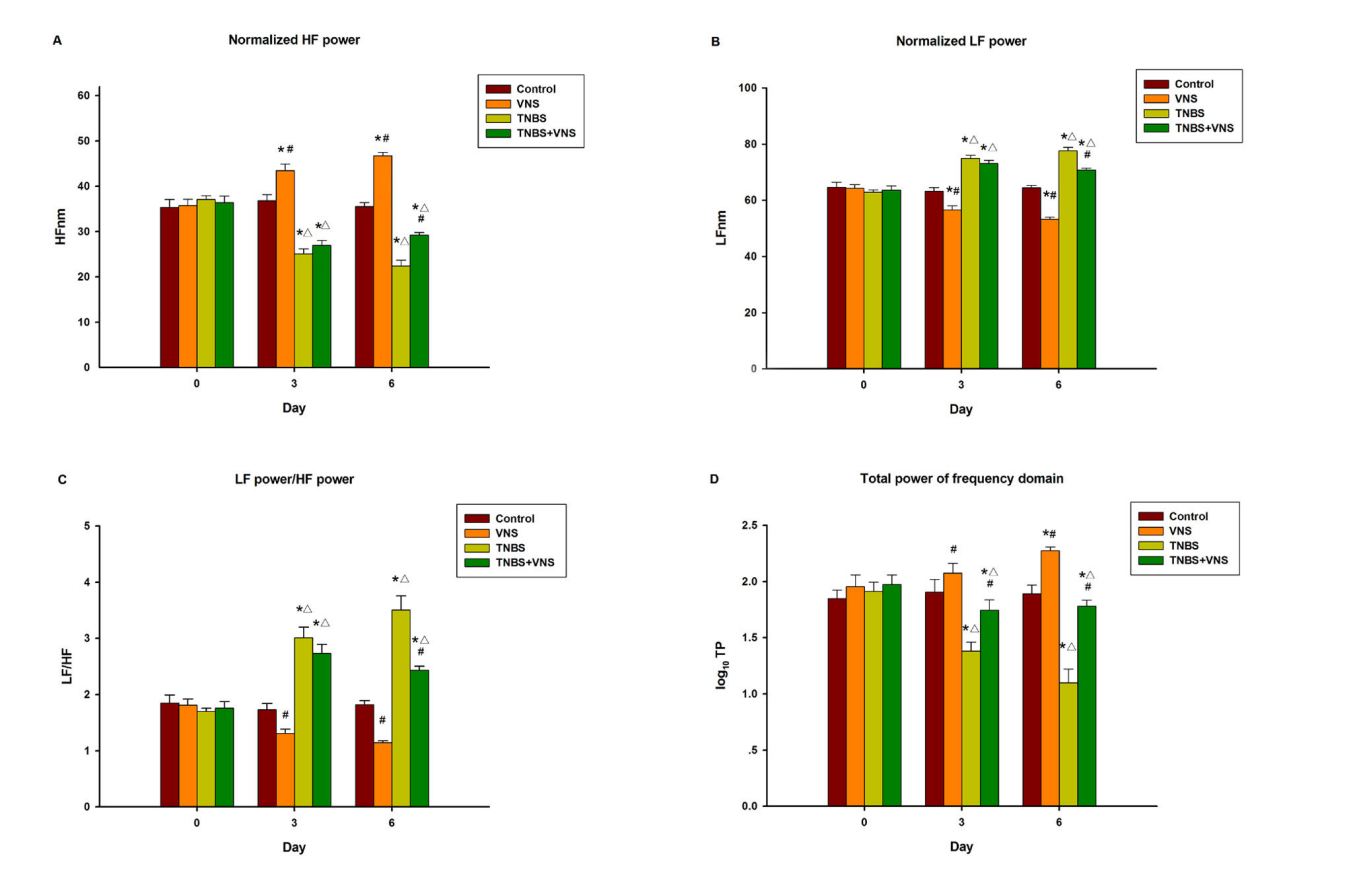
A–D. Effect of TNBS and VNS on LFnm(A), HFnm(B), LF/HF(C) and TP(D) on experimental colitis. * P<0.05 versus control group and VNS group; # P<0.05 versus TNBS group; △P<0.05 versus VNS group.

Figure **5A–D** shows the HRV parameters for the CON, VNS, TNBS and TNBS+VNS groups. LF/HF was significantly increased from baseline normalized low frequency (LFnm) values and from control group values in the TNBS group on day 3 and day 6 after TNBS injection (p<0.05). In contrast, TP and normalized high frequency (HFnm) values decreased significantly compared with basal levels and values in the control group (P<0.05) at the corresponding time points. In addition, a comparison between the TNBS and TNBS + VNS groups revealed that chronic VNS significantly inhibited the reduction of TP and HFnm on day 6 following TNBS injection (p<0.05), whereas LFnm and LF/HF were significantly decreased at the corresponding time point in the TNBS+VNS group (p<0.05). Furthermore, significantly decreased LFnm and LF/HF, as well as increased TP and HFnm, were observed in the VNS group at each time point compared with those in the basal and control groups (p<0.05).

### 3: Relationship between vagal heart rate modulation, TNF-α and IL-6 levels in TNBS-induced colitis

Table **2** presents correlations between HRV component (HFnm, LF/HF and TP) modulation and changes in pro-inflammatory factors (TNF-α and IL-6). After TNBS administration, IL-6 levels were significantly associated with HFnm (r=-0.512, p=0.004), LF/HF (r=0.554, p=0.001) and log _10_TP (r=-0.682, p<0.001). Similarly TNF-α levels were also associated with HFnm (r=-0.516, p=0.04), LF/HF (r=0.579, p=0.001) and log _10_TP (r=-0.591, p=0.001).

**Table 2:** Association between IL-6, TNF-α and HRV components.

	TNF-α		IL-6	
Variables	r	P	r	P
HFnm	-0.516	0.04	-0.512	0.004
LF/HF	0.579	0.001	0.554	0.001
log_10_TP	-0.682	<0.001	-0.591	<0.001

### 4: The possible mechanism of chronic VNS on TNBS-induced colitis

#### 4.1: Chronic VNS prevents activation of colonic NF-κB *in* *vivo*

**Figure 6:**
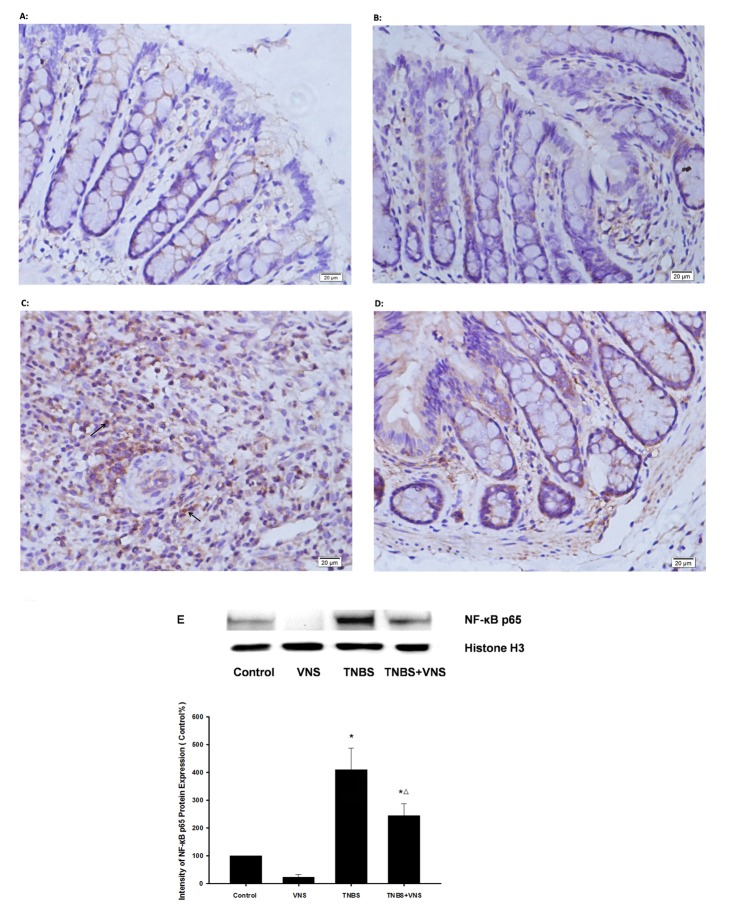
Chronic VNS inhibits the activation of NF-κB p65 on TNBS-induced colitis. Photomicrographs (magnification ×400) are representative of immunohistochemically stained slides with NF-κB p65 anti-body in colon mucosa. (A) and (B) show normal colon mucosa, and colon mucosa with TNBS (C) shows markedly increased NF-κB p65 nuclear-positive cells (arrow). Colon mucosa from the TNBS model treated with VNS (D) shows much less translocation of NF-κB p65. Western blot was also performed with NF-κB p65 anti-body (E), and densitometric analysis was normalized to Histone H3. The results are expressed as the mean ± SEM (n = 3). The data shown are representative of three independent experiments. * P<0.05 versus the control group; Δ P<0.05 versus the TNBS group.

Nuclear translocation of NF-κB has been identified as one of the most important signal transduction pathways involved in colitis. Therefore, we assessed the nuclear translocation of NF-κB’s p65 subunit in experimental colitis by immunohistochemistry staining and immunoblotting. As shown in Figure 6A–E, TNBS activated the expression of NF-κB p65 in colonic tissue, compared with the control group (p<0.01). However, treatment with chronic VNS in TNBS-induced colitis significantly reversed this effect, resulting in a pronounced reduction of NF-κB p65 expression (p<0.05).

#### 4.2: Acetylcholine inhibits the production of TNF-α and activation of NF-κB *in* *vitro*

**Figure 7:**
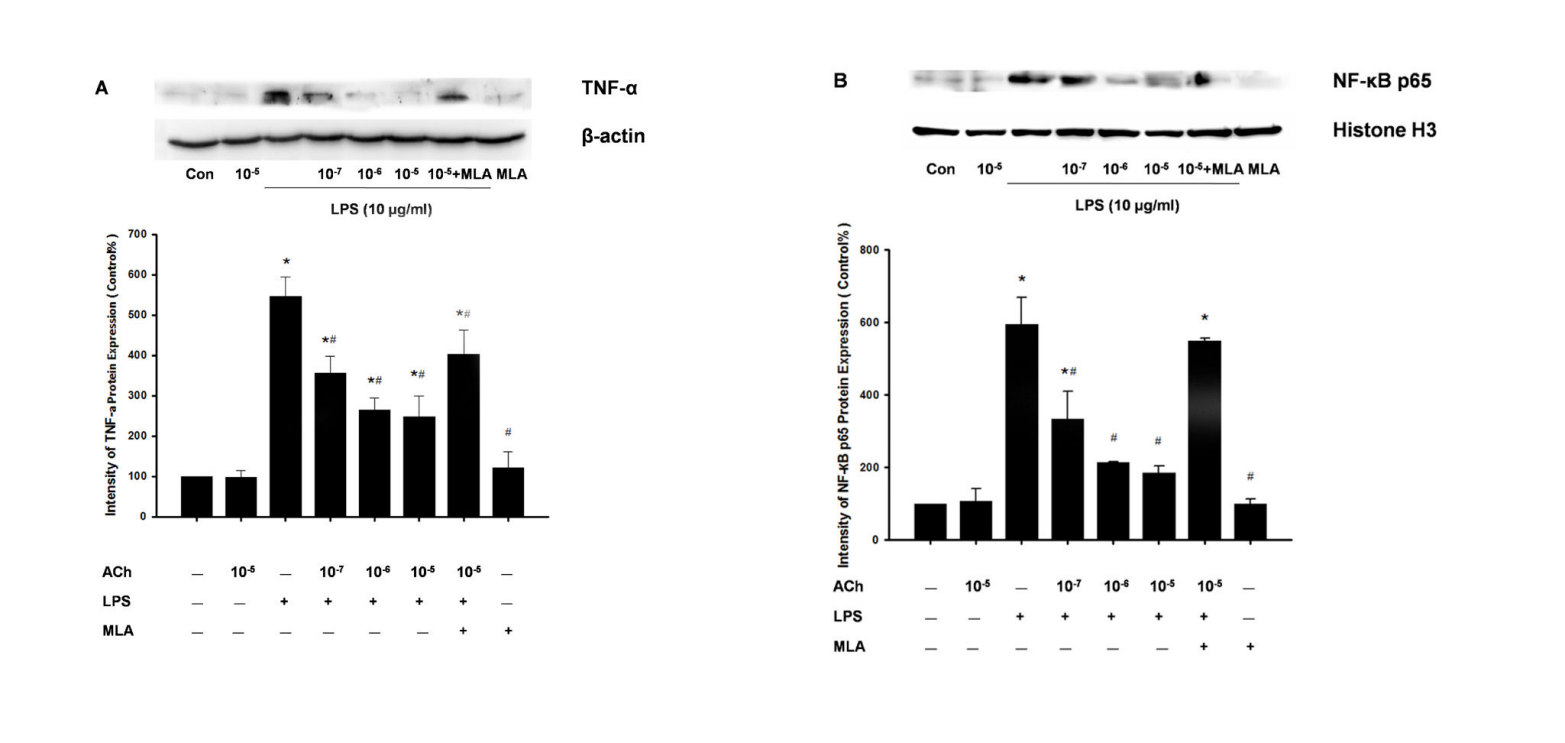
Acetylcholine inhibits LPS-induced TNF-α expression and NF-κB translocation in Caco-2 cells. Caco-2 cells (5×10^5^ cells/well) were pretreated with increasing concentrations of Acetylcholine (0.1-10 µM) with or without methyllycaconitine (10 µM) for 60 min and then incubated with or without LPS (10 µg/ml) for 24 h. Cells were then lysed, proteins from the whole cell were extracted, and the nuclear extract cells were analyzed by immunoblotting with anti-TNF-α antibody (A) and anti-NF-κB p65 antibody (B). Densitometric analysis was normalized to β-actin and Histone H3, respectively, and the results are expressed as the mean ± SEM (n = 3). The data shown are representative of three independent experiments. * P<0.05 versus the control group; # P<0.05 versus the LPS group.

The inhibitory effect of acetylcholine (ACh) on TNF-α and NF-κB p65 was also documented in cultured Caco-2 cells via immunoblotting. As shown in Figure **7**, expression of TNF-α and NF-κB p65 was significantly increased after 24 h of LPS stimulation in the Caco-2 cells compared with the control group (p<0.01). ACh at concentrations of 0.1-10 µM inhibited this TNF-α and NF-κB p65 activation in a concentration-dependent manner. However, as a specific antagonist of α7nAChRs, methyllycaconitine(MLA) only partly reversed the inhibitory effect of 10 µM ACh in LPS-stimulated Caco-2 cells (p<0.05).

#### 4.3: Chronic VNS suppresses phosphorylation of p38, ERK1/2, JNK MAPKs and IκB-α degradation *in* *vivo*

**Figure 8:**
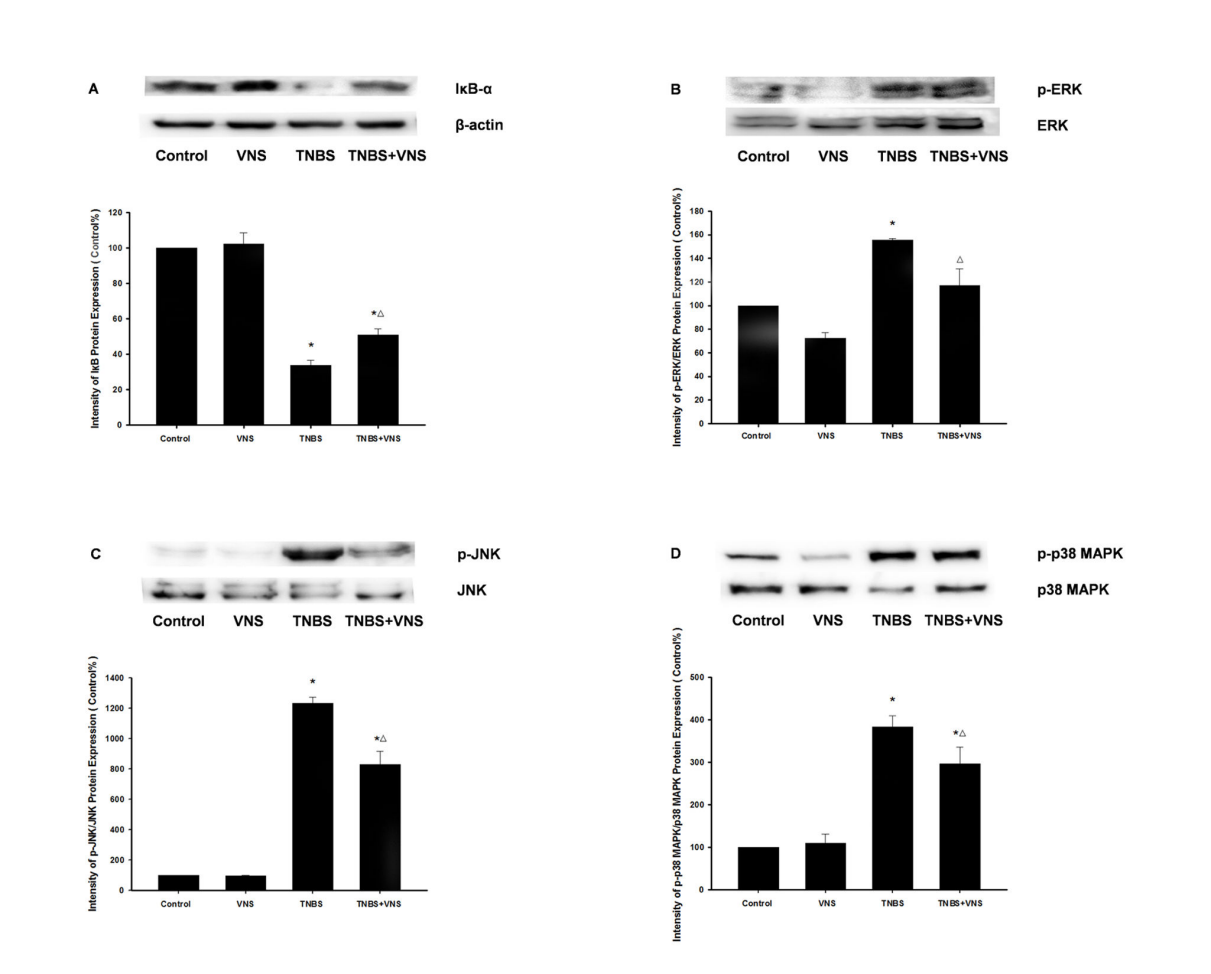
Chronic VNS inhibition of IκB-α (A) degradation and p-ERK1/2 (B), p-JNK (C), and p-p38 (D) activation in colon tissue from TNBS-induced colitis rats. Densitometric analysis was normalized to the control (ERK1/2, JNK, p38 and β-actin, respectively), and the results are expressed as the mean ± SEM (n = 3). The data shown are representative of three independent experiments. * P<0.05 versus the control group; Δ P<0.05 versus the TNBS group.

The effects of chronic VNS on TNBS-induced activation of the MAPK family (p38, ERK1/2, JNK) and IκB-α degradation, which are generally recognized as the upstream indicators of NF-κB signaling pathway activation, were evaluated by western blot. In the present study, as observed in Figure **8**, a high expression of phosphorylated p38, ERK1/2 and JNK was detected in cytosolic extracts of colon mucosa from TNBS-treated rats compared with the normal colon mucosa from the control group (p<0.01, p<0.05 and p<0.001, respectively), whereas treatment with chronic VNS significantly ameliorated the MAPK phosphorylation (p<0.01, p<0.05 and p<0.01, respectively), indicating that the administration of chronic VNS was able to diminish MAPK protein upregulation. Furthermore, TNBS-induced intestinal inflammation also resulted in a significant cytosolic IκB-α degradation that corresponded to the enhancement of NF-κB-binding activity, whereas VNS was able to block the TNBS-induced activation of the NF-κB pathway.

#### 4.4: Acetylcholine inhibits activation of the MAPK pathway *in* *vitro*

**Figure 9:**
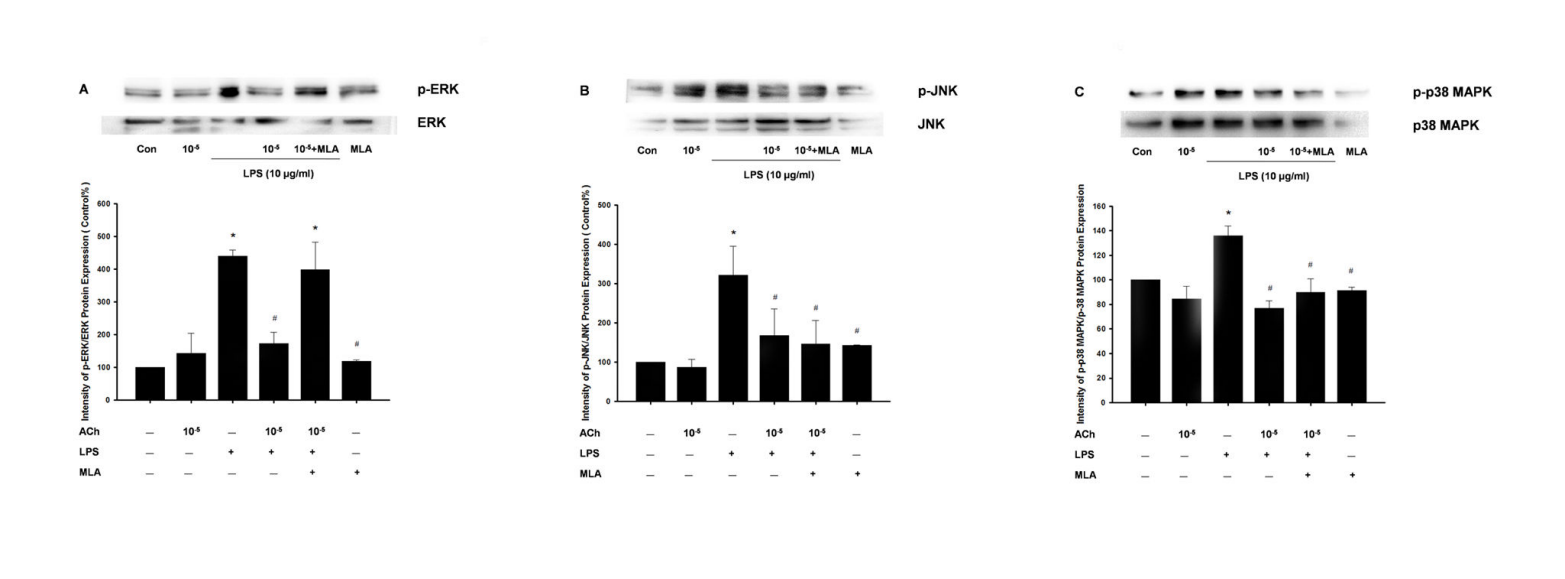
Acetylcholine inhibits LPS-induced activation of p-ERK1/2 (A), p-JNK (B) and p-p38 MAPK (C) in Caco-2 cells. Caco-2 cells (5×10^5^ cells/well) were pretreated with 10 µM Acetylcholine with or without methyllycaconitine (10 µM) for 60 min and then incubated with or without LPS(10 µg/ml) for 24 h. Cells were then lysed, and the proteins were analyzed by western blot. Densitometric analysis was normalized to the control (ERK1/2, JNK, and p38, respectively), and results are expressed as the mean±SEM (n = 3). The data shown are representative of three independent experiments. * P<0.05 versus the control group; # P<0.05 versus the LPS group.

The inhibitory effect of acetylcholine on the LPS-induced activation of the MAPK family was also evaluated in cultured Caco-2 cells by immunoblotting. Compared with the control group, the phosphorylation of p38, ERK1/2 and JNK was significantly increased after 24 h of LPS incubation in Caco-2 cells (p<0.01), whereas 10 µM ACh inhibited the activation of p38, ERK1/2 and JNK MAPK proteins (p<0.01, p<0.01 and p<0.02, respectively). In addition, MLA reversed the inhibitory effect of 10 µM ACh on the phosphorylation of ERK1/2 in LPS-stimulated Caco-2 cells (p<0.01). However, a similar MLA reversal was not observed in the phosphorylation of p38 and JNK in LPS-stimulated Caco-2 cells (Figure **9**).

## Discussion

Inflammatory bowel disease, which is characterized by chronic inflammation in the intestinal mucosa, affects millions of patients worldwide. Although the pathogenesis of IBD remains unclear, the dysfunction of intestinal immune regulation and an overproduction of pro-inflammatory cytokines by activated infiltrating macrophages are widely believed to be the mainstay of the disease initiation and perpetuation. However, emerging evidence suggests that the ANS dysfunctions, such as psychological disturbances, stress, depression, anxiety or negative psychological attributes, may have potential effects on the disease course of IBD by psycho-neuro-endocrine-immune modulation of the brain-gut axis, including the cholinergic anti-inflammatory reflex [30]. Given the ineffectiveness and the side effects of conventional drugs, many recent clinical trials have attempted to modify vagal efferent activity by device-guided VNS, biofeedback or aerobic exercise in order to demonstrate anti-inflammatory effects [31]. However, thus far, there has been no research delving into the mechanisms underlying these interventions for IBD.

In the present study, we established a chronic VNS model to demonstrate the possible anti-inflammatory molecular mechanisms of these interventions in relation to modified sympathetic-vagal balance on experimental colitis induced by TNBS. Normally, a low-frequency range of stimulation (1-5Hz) was used for investigating the anti-inflammatory effect of the activation of vagal efferents [32], however, vagal afferents may also be activated. A recent fMRI study of VNS have shown that even at a low frequency of stimulation (5Hz), a central effect was particularly found in the nucleus tractus solitarius (NTS) [33]. Moreover, the potential mechanism of CAM therapies such as biofeedback and relaxation training is thought to be central nervous system (CNS)-based, so a higher stimulus frequency(20Hz) used in the present study which involved the activation of both vagal afferents and efferents may preferably imitate CAM therapeutic effect on experimental colitis, and the significant amelioration of TNBS-induced colitis may be reflected by a combined activation of CNS(i.e. NTS-PVN-HPA axis) and CAP. TNBS was originally described to induce inflammation via haptenization of colonic mucosal components by Morris et al [34]. Inflammation induced by TNBS is not only confined to the colon mucosa and the lamina propria, but it also causes transmural ulceration. As a result, the TNBS model is supported as a suitable method for studying specific agents targeting biological processes, such as TNF signaling, cell junction organization, Interleukin-1 processing and fatty acid metabolism [35]. Our results demonstrated that chronic VNS significantly ameliorated the inflammatory symptoms of TNBS-induced colitis in rats, including weight loss, bleeding, and diarrhea, which manifested as remarkable decreases in the disease activity index (DAI) scores. As expected, the inspection of colon tissue sections and histological scores also indicated that vagal activation may be essential for the anti-inflammatory effects on experimental colitis.

Reactive oxygen and nitrogen species, which induce cellular injury, necrosis and remodeling of injured tissue, are predominantly derived from neutrophil activation in the colonic mucosa inflammation, and these factors contribute to the pathogenesis of IBD [36]. Here, our results demonstrated that the induction of colitis caused a significant increase in myeloperoxidase (MPO) activity, which predominantly existed in neutrophils, monocytes and macrophages, and is currently used as a quantitative index of oxidative stress in colonic mucosa [37]. Similar up-regulation of inducible nitric oxide synthase (iNOS) activity, which is described as a mediator of peroxynitrite formation, cell toxicity and activation of macrophages, was also observed in inflamed colonic mucosa. As expected, chronic VNS resulted in a strong reversal of these changes in the TNBS model. Given that activated neutrophils, monocytes and macrophages up-regulated the production of pro-inflammatory mediators such as TNF-α and IL-6 in experimental colitis, we further investigated whether chronic VNS could decrease the levels and expression of TNF-α and IL-6. In the TNBS model, VNS markedly decreased the production of both TNF-α and IL-6. The observations that the similar changes of the production TNF-a and IL-6 in the serum in systemic inflammation model indicated that VNS may reduce local or general release of pro-inﬂammatory cytokines directly or indirectly [38]. We also cultured Caco-2 cells in vitro and pre-treated (60 min) them with acetylcholine (0.1-10 µM) after LPS administration. The results demonstrated that, as the neurotransmitter of vagal efferents, acetylcholine induced a significant reduction of TNF-α expression in a concentration-dependent manner. In conclusion, these data indicate that improvement of vagal activity might modulate the activation of inflammatory immune cells and, consequently, might suppress oxidative stress and cytokine production.

We assumed that peripherally release of ACh by vagal efferents may modulate the activation of inflammatory immune cells via CAP and resulted in the amelioration of colitis. In the present study, we shown that chronic VNS significantly increased ACh level in colonic mucosa, however, there is currently no evidence that parasympathetic indeed innervate the distal colon. According to our results, a direct effect of ACh on colonic mucosal immune cells cannot be eliminated, but a growing number of studies implied that vagus nerve termini might not actually reaches the immune cells, the spleen and splenic nerve may play a role in mediating the anti-inﬂammatory effects of CAP [39]. Actually, ACh released by the vagal efferents may act on celiac-superior mesenteric ganglion to activate splenic nerve. The norepinephrine (NE) released by splenic nerve may interact with β-adrenergic receptor which is expressed on the acetylcholine-synthesizing T cells in white pulp [40,41] which revealed the possibility that the colonic ACh may be derived from splenic T cells. Whether or not peripherally release of ACh by vagal efferents acts on the colon directly, or indirectly, is currently under debate.

Sympathetic-vagal balance, which is thought to play an important role in the IBD disease course, was evaluated by the HRV frequency domain analysis. The frequency domain of HRV analysis displays two peaks, including a low frequency peak (LF) and a high frequency peak (HF). These peaks are frequently assumed to be predominantly mediated by cardiac sympathetic and parasympathetic neural activity, respectively, and therefore, the ratio of LF to HF is used to evaluate the balance between sympathetic and vagal modulation. The total power of the HRV frequency domain (TP) corresponds to the total amount of HRV, and a low or high TP may reflect a decreased or increased cardiac autonomic regulation, respectively [42,43]. The present study indicates that HF and TP were decreased after TNBS administration during experimental colitis, whereas the LF and LF/HF index were elevated. These data are also consistent with the clinical findings of autonomic dysfunction in IBD patients [14,15] and indicate that either the over-inhibition of the parasympathetic system and/or the over-excitation of the sympathetic system leads to disease progression. Additionally, we reveal that chronic VNS can regulate and rebalance the sympathetic-vagal balance, resulting in significant increases in HF and TP and decreases in LF and the LF/HF index within 6 days of TNBS administration. Moreover, both TNF-α and IL-6 levels were inversely correlated with HF and TP components. Based on our VNS model of TNBS-colitis, we believe that an improvement of vagal activity may rebalance and stabilize the autonomic system and suppress the occurrence and development of colon inflammation.

Previous reports indicated that the induction of the pro-inflammatory cytokines IL-1, IL-6 and TNF-α in colonic mucosa is mediated by intracellular signal transduction involving the NF-κB pathway and the activation of three MAP kinases (p38, ERK and JNK) [44,45]. Furthermore, recent studies presented the concept of a cholinergic anti-inflammatory pathway [18] through which peripherally released acetylcholine (ACh) by vagal efferents may suppress inflammatory responses by inhibiting NF-κB signaling via the α7 nicotinic acetylcholine receptor (nAChR) on immune cells [19,46]. However, growing evidence over the past decade integrated the anti-inflammatory effect of the VNS model in vivo and/or cholinergic agonists such as Ach or nicotine in vitro in IBD research [22-25,47], and the underlying mechanisms of this effect were rarely studied. The human colon carcinoma cell line caco-2 was commonly used in vitro to study the molecular mechanisms underlying differentiation, biosynthesis and drug absorption of intestinal epithelial cells, it may also be a suitable model to investigate colonic inflammatory immune response including pro-inﬂammatory cytokines release [48], intracellular activation of JNK and p38 MAPK signaling [49] as well as NF-κB translocation [50]. In this study, we show that phosphorylation of all three MAPKs was detectable in the TNBS-colitis model, and within 24 h after exposure of Caco-2 cells to 10 µg/ml of LPS, nuclear translocation of NF-κB p65 was also observed in vivo and in vitro. NF-κB is tightly bound to its endogenous inhibitor IκBα in the NF-κB- IκBα complex, and phosphorylated IκBα is subsequently ubiquitinated and degraded by the 26S proteasome [51]. NF-κB is then released and translocated to the nucleus [44]. As expected, after 6 days of chronic VNS, activation of ERK, p38 and JNK was also suppressed significantly and was accompanied with reduced IκBα degradation and NF-κB p65 translocation. Exposure to 10 µM ACh was also confirmed to down-regulate the activation of the MAPK family and the NF-κB pathway in LPS-stimulated Caco-2 cells. As mentioned above, the α7 nAChR is believed to be the main subtype of acetylcholine receptor that mediates the MAPK cascade [52,53]. Interestingly, as an α7nAChR antagonist, we found that methyllycaconitine only reversed the inhibition effect on p-ERK and intranuclear NF-κB p65 expression by 10 µM ACh in vitro, and no significant change was observed in the expression of p-p38 MAPK or p-JNK by methyllycaconitine incubation. This result suggests the possibility that other subtypes of nAChR may participate in the activation of the MAPK and NF-κB cascade, such as α5 nAChR [54]. Besides, a recent study by Cucina et al. demonstrated the anti-apoptotic and proliferative effect of nicotine on caco-2 cells and declared that PKC/ERK1/2 pathway may also be critical for colonic epithelial cells proliferation and survival and the involvement of α7-nAChR in this process [55], which indicating that activation of α7-nAChR may not only result in the suppression of inflammatory responses by CAP but also the proliferation and recovery process of injured epithelial cells by activation of MAPK cascade. Overall, we conclude that an improvement of vagal activity may activate ERK1/2 and NF-κB translocation, inducing the transcription of pro-inflammatory genes via an interaction of α7nAChR and peripheral release of ACh in inflamed colonic mucosa.

In summary, the present findings support the assumption that vagal activity modification may have a beneficial effect on IBD patients. Despite the fact that the general application of traditional VNS therapy in IBD is currently difficult to achieve, increasing clinical evidence has suggested that mind-body interventions or device-guided VNS can significantly enhance cardiovagal modulation by relaxation training and biofeedback [9]. We aim to demonstrate the possible anti-inflammatory molecular mechanism of these interventions on IBD by establishing this chronic VNS model, and clinical trials are warranted in IBD patients to explore if relaxation training and HRV-biofeedback can contribute to the improvement of clinical symptoms and the regulation of colonic inflammation.

## Materials and Methods

### 1: Experimental animals and ethics statements

Adult male and female Sprague-Dawley rats supplied by the Laboratory Animal Center, SUN YAT-SEN University, weighing 180-220g, were housed individually in cages under standard conditions: temperature 24-25°C, humidity 70-75%, lighting regimen of 12L/12D, and normal laboratory diets. Rats were deprived of food for 24 h prior to the induction of colitis but were allowed free access to tap water throughout. All animals were treated carefully in strict accordance with National Institutes of Health on animal care and the ethical guidelines, all experimental procedures were approved by the Animal Care And Use Committee of Sun Yat-sen University (Permit Numbers: SCXK(Guangdong) 2011-0029). All surgeries were performed under anesthesia and all efforts were made to minimize suffering.

### 2: Surgical procedures

Rats were anesthetized with 10% chloral hydrate (0.35 ml/100 g i.m). A horizontal incision was made in the ventral aspect of the neck. The skin and muscles were meticulously separated and the left vagus nerve which lies laterally to the carotid artery was exposed. The bipolar coil electrodes were placed around the left cervical vagus nerve and the left carotid artery, and it was ensured that the electrodes were in close contact with the vagus nerve [56,57]. The electrodes were linked to a connector fixed to the head with dental cement, and the connector was linked to a stimulator (BL-420, Tme Technology Co., Ltd, Chengdu, China). Sham animals underwent the same surgical procedure with leads and the stimulator. The rats were injected with penicillin immediately after surgery, and topical antibiotic was applied to the wound.

### 3: Animal experiments

Thirty-six rats were previously randomized into four groups: sham-VNS/saline injected (controls, n=8); VNS/saline injected (VNS, n=8); sham-VNS/TNBS injected (TNBS, n=10); and VNS/TNBS injected (VNS+TNBS, n=10). Seven days after surgery, rats deprived of food for at least 24 h were anesthetized with 10% chloral hydrate (0.35 ml/100 g i.m) at experimental day 0. They then received an intrarectal administration of 2,4,6-Trinitrobenzenesulfonic acid (TNBS, 250 ml, 150 mg/Kg, Sigma-Aldrich, Saint-Louis, USA) dissolved in a 1:1 mixture of 0.9% NaCl with 100% ethanol. Control rats received a 1:1 mixture of 0.9% NaCl with 100% ethanol or a saline solution using the same technique described by Morris et al [58]. Rats were maintained in the head-down position for 10 min following intracolonic administration. The rats received VNS for 3 h per day from experimental day 1 to day 6 using standard stimulation parameters (0.25 mA, 20 Hz, 500 ms pulse width, 30 s ON, 5 min OFF continuously) [59]. Rats in control group were exposed to the same procedure but did not receive the stimulation.

### 4: Heart rate variability (HRV)

The lead II electrocardiogram was recorded at experimental day 0, day 3 and day 6, and the HRV components were analyzed by zA Spirit Nexus-16B (Spirit-Ming, Netherlands) and its software (Biotrace + version 1.20, Mind media B.V. Netherlands). HRV, assessed in the frequency domain of the power spectrum in RR intervals, was computed by fast Fourier transforms on the sinus consecutive R wave intervals (RR intervals) obtained from the 15 min observations at each time point. The frequency domains of HRV were classified as follows according to Lo Giudice et al [60]: (1) very low frequency (VLF): 0.025–0.199 Hz; (2) low frequency (LF): 0.20–0.59 Hz; (3) high frequency (HF): 0.60–2.5 Hz; and total power (TP): 0.00–2.5 Hz. The normalized LF and HF were calculated as follows: LF (HF) nm = LF (or) HF/TP–VLF×100%. To eliminate the impact of changes of HRV at different times of the day, rats were examined at the same time on each experimental day.

### 5: Quantification of disease activity

Body weights of rats were measured daily, from day 0 to day 6, and the clinical disease activity index (DAI) was measured daily using the protocol previously described (Table **3**) [61]. The scores from these three parameters were summed as the DAI, ranging from 0 (healthy) to 12 (maximal severity of colitis).

**Table 3:** Scoring of disease activity index (DAI).

Weigh loss	Stool consistency	Bleeding
0: <1%	0: normal	0: negative
1: 1-5%	2: loose stools	2: positive
2: 5-10%	4: diarrhea	4: gross bleeding
3: 10-15%		
4: >15%		

To determine the DAI, scores for weight loss, stool consistency and bleeding were measured daily. Weight loss was calculated as the percentage difference between the body weight on day 0 and every other day of the experiment. Diarrhoea was defined as mucus/faecal material adherent to fur and bleeding was detected by occult blood test [37].

### 6: Macroscopic evaluation of colonic damage

All rats were sacrificed on experimental day 6. The colon was removed from the cecum to anus, opened longitudinally and rinsed with cold physiological saline to remove fecal residues. The colon was examined visually, and damage was assessed by the Colon Mucosal Damage Index (CMDI) scored on a scale of 0-10 according to the criteria described by Tsune et al [62]. The following scores were applied: Grade 0: normal appearance; Grade 1: focal hyperemia, no ulcers; Grade 2: ulcer with no significant inflammation (hyperemia and bowel wall thickening); Grade 3: ulcer with inflammation at one site; Grade 4: 2 or more sites of ulceration and/or inflammation; Grade 5: major site(s) of damage extending >1 cm along the length of the colon; and Grade 6-8: when an area of damage extended >2 cm along the colon, the score was increased by 1 for each additional cm of involvement.

### 7: Microscopic evaluation of colonic damage

Representative colon tissue samples were fixed in 4% paraformaldehyde in PBS overnight. The formalin-fixed colon tissues were embedded in paraffin wax, and 5 µm specimens were subjected to hematoxylin and eosin (H&E) staining for pathomorphological examination. The following histological parameters were studied: for inflammatory infiltrate, grading was considered as severe=3, moderate=2, mild=1, absent=0; for inflammatory infiltrate, grading was considered as severe=3, moderate=2, mild=1, absent=0; for ulcers, grading was considered as diffuse glandular disruption or extensive deep ulceration=4, glandular disruption or focal deep ulceration=3, diffuse superficial ulceration=2, focal superficial ulceration=1, absent=0 [63]. The total colitis inflammatory index was then derived by summing 3 subscores (ulceration, hyperplasia, and inflammatory infiltrate) on H&E-stained tissue.

### 8: Measurement of myeloperoxidase (MPO) activity and inducible nitric oxide synthase (iNOS), ACh levels

Tissue samples (1 g) were homogenized in 10 ml of ice-cold physiological saline. The homogenate was processed to measure MPO activity (Nanjing Jiancheng Biochemical Engineering, Nanjing, China) as described by Yegen et al. [64] according to the O-dianisidine method. The MPO activity of colonic samples was determined as an index of neutrophil infiltration in the mucosa. iNOS levels have also been considered a determinant of colonic damage, and the homogenate mentioned above was also processed for the detection of iNOS levels (Nanjing Jiancheng Biochemical Engineering, Nanjing, China) according to the method described by Ryoyama et al [65]. The concentration of colonic acetylcholine (ACh) (Nanjing Jiancheng Biochemical Engineering, Nanjing, China) was detected using the method of Hestrin, as described by Patil [66].

### 9: Tumor necrosis factor-alpha (TNF-α) and Interleukin-6 (IL-6) in colon tissue

Mid-colon segments were homogenized and centrifuged at 3,000×g for 10 min, and the supernatants were used for the determination of cytokines levels. TNF-α and IL-6 levels in colon lysates were analyzed by sandwich enzyme-linked immunosorbent assays (ELISA) using an ELISA kit according to the manufacturer’s instructions (Raybiotech, Georgia, USA).

### 10: Immunohistochemistry analysis of the expression of NF-κB (p65) in colon tissue

Colon paraffin-embedded sections of 0.5 µm thickness were de-waxed and rehydrated through graded concentrations of ethanol. After 30 min of antigen retrieval with a sodium citrate buffer (0.01 mol/L, pH 6.0) by microwaving, the slices were incubated with 2% hydrogen peroxide for 10 min to block the endogenous peroxidase. Bovine serum albumin was applied to block the non-specific binding for 30 min. Then, slices were incubated with the NF-κB (p65) antibody (1:50, #4764S, Cell signaling Technology) at 4° C overnight. After washing 3 times in PBS for 5 min each time, slices were incubated with biotin-labeled secondary antibody (1:100) for 60 min at room temperature. The chromogen DAB was applied for color reaction, which was monitored under the microscope and terminated with distilled water. Counter-staining was visualized by hematoxylin for 60 s, and the slices were then dehydrated and cover slipped with neutral gum. Immunohistochemistry was visualized using an Olympus BX41 microscope and recorded with a high-resolution DP70 Olympus digital camera. Pictures were photographed.

### 11: Cell Cultures and Treatments

Caco-2 cells were obtained from Cell Bank, Chinese Academy of Sciences (www.cellbank.org.cn), and cultured in Dulbecco’s Modified Eagle’s Medium (DMEM) supplemented with 10% fetal bovine serum, 100 U/ml penicillin, 100 µg/ml streptomycin, and maintained at 37° C in a humidified incubator containing 5% CO_2_ (changing the medium every other day). The cells were seeded in 6-well cell culture plates at 5×10^5^ cells/well and cultured to reach 80% confluency.

For the treatments, cells were treated with LPS from *Escherichia coli* 055:B5 (10 µg/ml, Sigma-Aldrich, Saint-Louis, USA) for 24 h. Before LPS stimulation, some wells were pre-treated with different concentrations (0.1-10 µM) of acetylcholine (Sigma-Aldrich, Saint-Louis, USA) or methyllycaconitine (10 µM, Sigma-Aldrich, Saint-Louis, USA), which is a specific antagonist of α7nAChRs. After 1 h of incubation at 37° C, cell cultures were then stimulated with LPS as previously described. Untreated cells were used as controls.

### 12: Immunoblotting

Colon tissue or Caco-2 cells were collected and lysed by an ultrasonic cell disruptor or homogenizer in lysis buffer (#9806S, Cell signaling Technology) and incubated for 30 min on ice, centrifuged at 4° C for 10 min and stored at -20° C as the whole cell lysates. Nuclear extracts were prepared with a nuclear extract kit (#40010, Active Motif) according to manufacturer’s instructions.

The whole cell lysates were prepared for the evaluation of p-ERK 1/2 (1:1000, #4370S, Cell signaling Technology), ERK 1/2 (1:1000, #4695S, Cell signaling Technology), p-p38 MAPK (1:1000, #4511S, Cell signaling Technology), p38 MAPK (1:1000, #8690S, Cell signaling Technology), p-JNK (1:1000, #9258S, Cell signaling Technology), JNK (1:1000, #9255S, Cell signaling Technology), IκB-α(1:1000, #4814P, Cell signaling Technology), TNF-α(1:1000, #6945S, Cell signaling Technology) and β-actin (1:2000, #ACTBD11B7, Santa Cruz Biotechnology, Santa Cruz, CA, USA) expression. Furthermore, nuclear extracts were prepared for the evaluation of NF-κB p65 (1:1000, #4764S, Cell signaling Technology) activation and Histone H3 (#D1H2, Cell signaling Technology) expression by immunoblotting. Lysates were separated by SDS-PAGE, and primary and secondary Abs were incubated with the membranes with standard techniques. Immunodetection was accomplished using enhanced chemiluminescence. Chemiluminescence was acquired with a quantitative digital imaging system (Quantity One, BioRad, Hercules, CA), allowing a check for saturation. Overall emitted photons were quantified for each band, particularly for loading controls, which were homogeneously loaded. All western blot analyses were carried out in triplicate.

### 13: Statistical analyses

The results are presented as the mean ± standard error of the mean (mean ± S.E.M). Log_10_ transformations were made to TP to correct for skewness. Statistical analysis was performed with SPSS 13.0 statistical software. Macroscopic and microscopic data were compared with the Kruskal-Wallis nonparametric test, and other parameters were compared by one-way ANOVA with post-hoc testing by Tukey’s method. Associations between HFnm, LF/HF, log _10_TP and TNF-α and IL-6 levels were evaluated using correlations, and the differences were considered significant when p< 0.05.
